# Guy's and the Southwark Guardians

**Published:** 1912-11-02

**Authors:** E. C. Perry

**Affiliations:** Superintendent. Guy's Hospital, London, S.E.


### Guy's and the Southwark Guardians.

We have received a copy of the following letter:—

Sir,—The cases referred to in your letter of the 18th instant seem to be precisely similar in character to those which have on several former occasions occupied the attention of your Board and the authorities of Guy's Hospital, and, strictly speaking, no reply is required other than to refer you to previous correspondence. As, however, some of your members may not be familiar with the long history of the controversy, and may fail to realise how often your Board has been reminded of the duty it owes to the public and the ratepayers of Southwark, the House Committee direct me to set out for their information extracts from its own minutes, from the press, and from communications addressed to your Board at various intervals since the time when, on the passing of the Government Act of 1899, Guy's was transferred from the St. Olave's Union to the Poor-Law area administered by the Southwark Guardians.

As long ago as 1902 the house physician and house surgeon on duty complained that urgent cases sent by them to the workhouse of the Southwark Union had not been admitted to the infirmary.

In January 1904 the Clerk to your Board called attention to the case of Alfred R. The Committee fully considered the circumstances, and decided that no blame attached to the medical officers concerned. A copy of the comments of the *Guy's Hospital Gazette* upon this case is enclosed herewith.

In March 1904 a statement was read by a house surgeon with regard to the case of a man who alleged that, though in a desperate condition, he had been refused admission to the workhouse of the Southwark Union in the Marshalsea Road. The superintendent was directed to forward the house surgeon's statement to the Local Government Board, with a request that they would investigate the case.

Early in August 1905 a letter was written by the Clerk of your Board relative to the case of Louisa G., which was considered by the House Committee at their first meeting in September, and copies of the reply made to your Board were sent to King Edward's Hospital Fund for London, the Hospital Sunday Fund, and the Local Government Board. A communication was received by the Hospital from King Edward's Fund at the end of September to the effect that the Executive Committee "considered that the charge made against Guy's Hospital has been completely answered," and the letter was acknowledged without comment by the Local Government Board and the Hospital Sunday Fund.

I am instructed to quote the relevant portions of the reply then made to your complaint, which appear to be equally applicable to the cases now brought to the notice of the House Committee.

"So far as the resources of this Hospital go, they are always at the disposal of the sick poor, but it must be recognised that those resources are, so far as in-patients are concerned, strictly limited to 602 beds and cots. Whilst most anxious to render the Hospital of the utmost service to those around them, the Governors are compelled to keep within the limits of their accommodation; on the other hand, the Guardians have by law imposed upon them a responsibility limited only by the necessities of the poor within their Union.

"As large ratepayers, the Governors regard it as a matter which concerns them as well as the public that the Southwark Guardians should fail to provide infirmary accommodation within the limits of their Union. It is this failure on their part to equip a proper outpost hospital (reception station) within their boundaries which is the real cause of the trouble that occurs from time to time; and to place blame upon Guy's Hospital is to ignore responsibilities which, when the attention of the ratepayers is once aroused, cannot long be disregarded. To quote from Dr. P.'s report: 'Louisa G., if sent to the infirmary, would very likely have died on the journey.' No more serious charge could well be made against the Poor-Law authorities of Southwark. It is as much as to say that the sick poor must remain in their miserable homes, or must put up with workhouse treatment, if at the time of their mortal need they are too ill to bear transference to an infirmary 'four miles distant.' Such a lamentable condition of affairs should attract the immediate attention of your Guardians and of the Local Government Board, to whom a copy of this correspondence will be sent."

In November of last year public attention was directed to the case of a woman, Mrs. B., who, when there was not a single empty bed at Guy's for a female erysipelas case, was referred to yourselves, as the Poor-Law authority, and who died in your infirmary. In reply to the inquiries of a subscriber a letter was written, and afterwards published in The Hospital newspaper, from which extracts may be quoted :—

"Under these circumstances, having reached the utmost limit of our accommodation, what were we to do? Obviously it remained to call upon the Guardians to perform their duty to the sick poor to provide them with effective medical attendance, and this is where they fail to realise their own shortcomings, as is well pointed out in The Hospital newspaper; but it should be added in fairness to them that it is only since the passing of the London Government Act in 1899 that Guy's Hospital has been in the Southwark Union, and up to that time the people of Southwark had all the benefits and none of the inconveniences of a large London hospital in their midst. Hence a certain natural reluctance to modify their old arrangements, though they no longer suit the changed conditions, and though Guy's, in addition to saving the Union large sums in outdoor medical relief, is now paying the poor rate upon a heavy assessment of many thousand pounds.

"You may hardly be aware how complicated are the arrangements with the Poor-Law officers of Southwark. Our doctors have no authority to send a patient direct to the infirmary, however ill he may be. He has to go to the Relief Offices in the Borough Road, and these offices are closed between 12 noon and 3 p.m. After 5 o'clock in the evening patients have to go to one or other workhouse—men to Mint Street and women to Westmoreland Road, and it sounds incredible, but it is true, that the Guardians have no complete arrangements at either place for cases, whether from Guy's or elsewhere, so ill as to require the comforts of a sick ward, or so much injured that they cannot safely be taken three miles by ambulance to Dulwich. Their argument is that such cases ought never to be sent to them; our reply is that we never do send them until the limit of our available accommodation is reached, and that it is then their part to do what is obviously demanded—namely, to provide emergency beds and proper medical and nursing attendance, centrally in their own area for cases that cannot be moved to Dulwich, and, for such cases as can, to have an efficient ambulance service at all hours of day and night."

With these evidences of their own defective arrangements again brought to their knowledge it may be hoped that the Guardians of Southwark will take steps to put their own house in order, for assuredly in the minds of all thinking men and women, whatever of "scandal and disgrace to South East London" attaches to these unhappy incidents lies at their door, and not with the Governors of this Hospital, who, so far as their resources and opportunities permit, endeavour to make the great institution committed to their charge of the widest possible benefit to the sick and suffering. Having done their part, they expect the Guardians of Southwark to supplement the efforts of Guy's, and to fulfil the obligations laid upon them alike by law and humanity.—I am, dear Sir, yours faithfully,

(Signed) E. C. Perry, Superintendent.

Guy's Hospital, London, S.E.,
October 25.

The Clerk to the Guardians,
Southwark Union, S.E.

